# Supplementary material for: Expanding or shrinking? range shifts in wild ungulates under climate change in Pamir-Karakoram mountains, Pakistan
Source: PLoS One. 2021 Dec 31;16(12):e0260031. doi: 10.1371/journal.pone.0260031 (PMC8719741; doi:10.1371/journal.pone.0260031)
Supplement: S1 Map — (DOCX) [file pone.0260031.s001.docx]

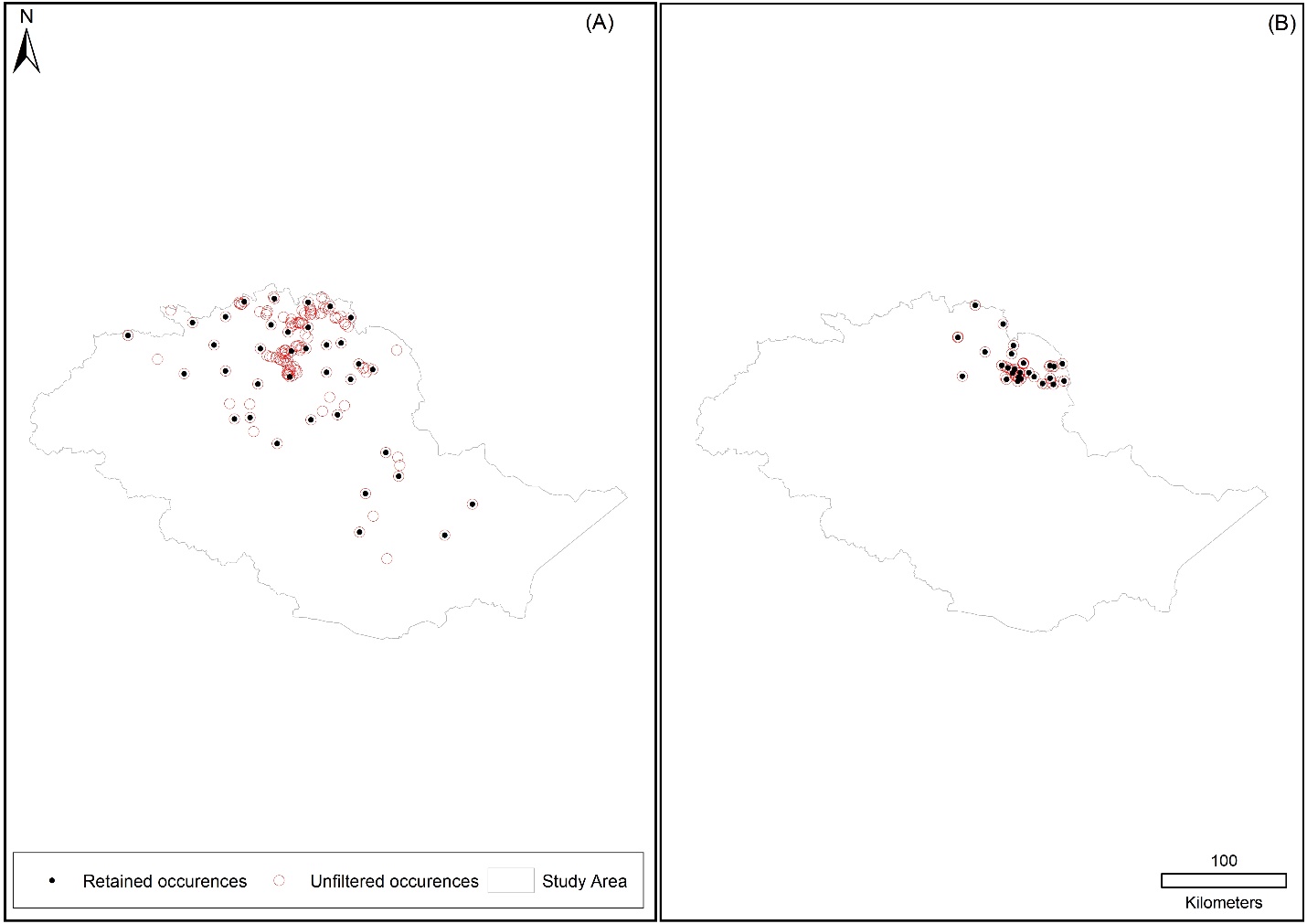


**S1**: Map showing unfiltered and retained occurrences used for the current study A) Himalayan ibex (total 143 points, retained points 36) B) Blue sheep (total 60 points, retained points 29) using SDMtoolbox V1.1(Brown 2014).
