# Supplementary material for: Expanding or shrinking? range shifts in wild ungulates under climate change in Pamir-Karakoram mountains, Pakistan
Source: PLoS One. 2021 Dec 31;16(12):e0260031. doi: 10.1371/journal.pone.0260031 (PMC8719741; doi:10.1371/journal.pone.0260031)
Supplement: S2 Table — (DOCX) [file pone.0260031.s003.docx]

**S2 Table**: Estimates of relative contributions of the environmental variables used to build MaxEnt model for Himalayan ibex

| **Variable** | **Percent**  **contribution** | **Permutation**  **importance** |
| --- | --- | --- |
| bio_12 | 42.7 | 56.6 |
| bio_01 | 23.2 | 24.4 |
| bio_15 | 23.2 | 7.5 |
| bio_04 | 5.8 | 4.8 |
| bio_08 | 3.3 | 2.7 |
| bio_02 | 1.8 | 4.1 |
