# Supplementary material for: Expanding or shrinking? range shifts in wild ungulates under climate change in Pamir-Karakoram mountains, Pakistan
Source: PLoS One. 2021 Dec 31;16(12):e0260031. doi: 10.1371/journal.pone.0260031 (PMC8719741; doi:10.1371/journal.pone.0260031)
Supplement: S7 Fig — Negative values indicate novel climate in the MESS map across the range. b) Most dissimilar variables (MOD) analysis shows those novel climatic conditions and the associated variables. (DOCX) [file pone.0260031.s010.docx]

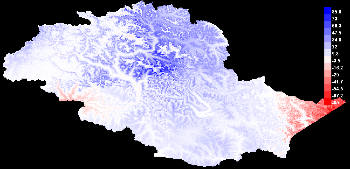

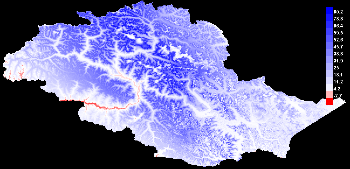

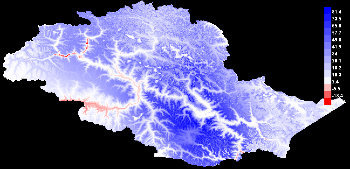

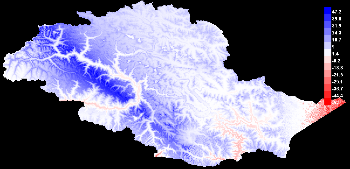


MESS (Multivariate Environment Similarity Surface)


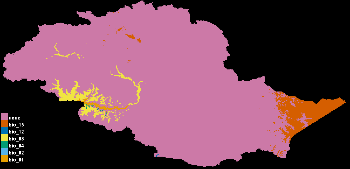

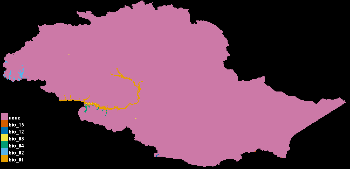

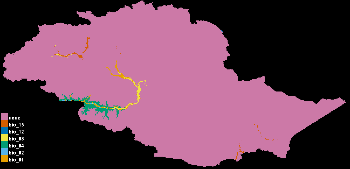

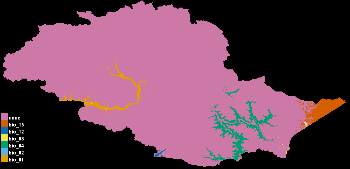


MoD (Most dissimilar Variable)

**S7 Fig:**Maps illustrating multivariate environmental similarity surface (MESS) approach as described in(Elith et al. 2010) and the most dissimilar variables(MOD) for Blue sheep under the year 2070 Representative Concentration Pathway (RCP4.5) for different Global Circulation Models. Negative values indicate novel climate in the MESS map across the range. b) Most dissimilar variables (MOD) analysis shows those novel climatic conditions and the associated variables.
