# Supplementary material for: Expanding or shrinking? range shifts in wild ungulates under climate change in Pamir-Karakoram mountains, Pakistan
Source: PLoS One. 2021 Dec 31;16(12):e0260031. doi: 10.1371/journal.pone.0260031 (PMC8719741; doi:10.1371/journal.pone.0260031)
Supplement: Attachment — Submitted filename: Repsonses to Reviewers_AN.docx [file pone.0260031.s012.docx]

**Response to Reviewers Comments**

Tzen-Yuh Chiang
Academic Editor
PLOS ONE

Dear Dr Chiang,

Thanks for completing review process of our MS (PONE-D-21-17916) “Expanding or shrinking? range shifts in wild ungulates under climate change in Pamir-Karakoram Mountains, Pakistan”, and allowing us to submit a revised version.
We have carefully addressed each and every comment, and details of changes are provided below. We thank the anonymous reviewer and editor for very insightful comments and helping Improve the MS.

We are including following items with our revised submission:

- A rebuttal letter that responds to each point raised by the academic editor and reviewer(s).
- A marked-up copy of your manuscript that highlights changes made to the original version.
- An unmarked version of your revised paper without tracked changes.

**Best Regards**

**Muhammad Ali Nawaz,** PhD

Associate Professor,

Department of Biological and Environmental Sciences

College of Arts and Sciences

**Qatar University**

**Journal Requirements:**

When submitting your revision, we need you to address these additional requirements.

1. Please ensure that your manuscript meets PLOS ONE's style requirements, including those for file naming. The PLOS ONE style templates can be found at
   <https://journals.plos.org/plosone/s/file?id=wjVg/PLOSOne_formatting_sample_main_body.pdf> and 
   <https://journals.plos.org/plosone/s/file?id=ba62/PLOSOne_formatting_sample_title_authors_affiliations.pdf\>

Response: MS is prepared according to the PLOS ONE's style requirements.

2. We note that the grant information you provided in the ‘Funding Information’ and ‘Financial Disclosure’ sections do not match.

When you resubmit, please ensure that you provide the correct grant numbers for the awards you received for your study in the ‘Funding Information’ section

Response: Necessary correction made

3. We note that Figure(s) 1, 2, 3, 4, 5, 6, and 7 in your submission contain map images which may be copyrighted. All PLOS content is published under the Creative Commons Attribution License (CC BY 4.0), which means that the manuscript, images, and Supporting Information files will be freely available online, and any third party is permitted to access, download, copy, distribute, and use these materials in any way, even commercially, with proper attribution. For these reasons, we cannot publish previously copyrighted maps or satellite images created using proprietary data, such as Google software (Google Maps, Street View, and Earth). For more information, see our copyright guidelines: <http://journals.plos.org/plosone/s/licenses-and-copyright>.

Response: These maps are outputs of the current study and not taken from any source. Copyright lies with the authors

**Reviewers' comments:**

Reviewer #1: In general, an interesting item and approach. Some information missing as regards the field methods used and the selected parameters with a predictive value. The number of figures should be reduced and figures should be enriched with boundaries (main parks, major mountain systems). Several inaccuracies found across MS, as follows:

Line 63: Moschus instead of Mouchus

Response: The suggested change is made in the line 63.

Lines 75-76: please rephrase in something like: “It was found in relatively aris precipitous mountain ranges, living well above the tree line at elevations of 3500-5000 m.”

Response: The lines have been rephrased as suggested but “the sentence is still kept in present tense” as the condition still applies. In lines 75 and 76.

Lines 77-81: please rephrase in something like: “On the other hand, the blue sheep or bharal, an intermediate wild caprine species between the goat and the sheep, is found in less precipitous areas compared with ibex, at altitudes of 3500-5500 m in slopes covered with grasses and sedges.” Low (or gentle) slopes may be confusing

Response: The lines have been rephrased as suggested, please check in lines 77 – 82.

Lines 88-89: please rephrase in something like: “Currently, wild ungulate distribution in Gilgit-Baltistan (GB) is only partially known, and knowledge of climate-change induced impacts on species and habitats is insufficient.” The distribition of wild ungulates is locally very well known (e.g., within CKNP)

Response: The lines have been rephrased as suggested. Please check in lines 89-91.

Line 110: use “rare” and delete “endangered”

Response: The word “endangered” deleted in line 110.

Line 121: “in all potential areas” is a vague expression. Please be more accurate in describing the criteria underpinning the positioning of cameras.

Response: “in all potential areas” is replaced by “for” in line 121.

Line 122: substitute “installed” with “operated” or something similar

Response: “installed” is replaced by “they were left operational for” it is now in line 125.

Line 122: add details on the season/s when cameras where positioned. In addition, explain the reasons for such a wide range (10-40) in camera operation days.

Response: We have added suggested changes and repharsed the paragraph as “Camera trapping: We installed 225 (Reconyx HC 500 and HC 900; Reconyx, Holmen, USA) cameras during the period 2010–2016 for *C. ibex sibirica* and *P. nayaur*, in different months of the year i.e., Khunjerab National Park (KNP) (November to January, 2010 and September to November, 2011), in Qurumber National Park (QNP) (May to June 2012) in Misgar Valley (May to July, 2013), in Hopper and Hisper Valleys (March to May, 2016). Cameras were left operational for 10 days in the first camera trapping in KNP, but in the latter surveys they were left operational for 40 days to increase capture rate [42,43].” Please check this in lines 122 – 127.

Lines 123-128: please, help the reader understand the reason/s for use of a “double observer survey” approach for the purposes of this study.

Response: The following lines have been added to clear the reader about the locations of survey: lines 128-129

We carried out this survey in 2012–2016 in different parts (KNP, Gojal Valley, Shigar Valley, in Skardu district, and in Gilgit district) of the study area.

and to elaborate purpose of double observer method “The locations where Himalayan ibex and blue sheep were sighted, have been used as presence points to build the MaxEnt model” please see these changes in lines 134 – 135.

Line 129: what do Authors exactly mean as “records”? Please define

Response: The “records” means presence points, but now we have replaced “records” with presence points for clear understanding of reader. In lines 136,137 and 141.

Lines 167-175: There is something missing in this phrase (like some “.”or “,”). Please review with accuracy.

Response: We have now cleared the lines for easy understanding of readers as “The model was projected to entire GB. To project the models calibrated for survey area over entire GB, the variables in the projection area must meet a condition of environmental similarity with the environmental data used for calibrating the model. Therefore, we preliminarily ascertained that this condition was verified for both current and future projections by inspecting Multivariate Environmental Similarity Surfaces (MESS), the MESS calculates the similarity of each point in the region of projection to a set of reference points (e.g., background data) and maps the results [56] MESS maps produced by MaxEnt can help users identify extrapolated areas and provide a quantitative measure of projection uncertainty.” In lines 177 – 179.

Lines 213-214: Fig. 2 should be more informative about the limits between Himalaya, Hindu Kush and Karakoram

Response: We have added required information in the map in lines 220 – 221.

Lines 212-223: Authors should be more explicit on the significance of identified predicting parameters for ibex and blue sheep (e.g., what does exactly mean “precipitation seasonality”? According to analyses, which season should be the rainmost in a blue sheep suitable range?). I think a dedicated table is necessary and I’m sure that readers would appreciate

Response: Thank you very much for your precious comments. We have added following phrase to clarify this point (please, see lines 147 - 148).

Further details and information on the realization and interpretation of the WorldClim variables used in this study can be found at <https://pubs.usgs.gov/ds/691/>.

Lines 293-240: Please, add what does it mean in terms of % reduction of suitable surface.

Response: We have added percentage of reduction in the suggested lines e.g., 247 and 249.

Lines 241-243: Table 2 is not clear. Do numbers refer to pixels? For the sake of clarity, please add an additional right column with “TOTAL”.

Response: Yes, the numbers are pixels, we have added a table “titled as Total” as suggested in lines 252 – 253.

Line 283: insert something like “although at the same time thinning” after “expanding”. This is very important to stress

Response: We repharsed the line as “watersheds of the Karakoram are expanding [71] although at the same time they are thinning” in line 294.

Line 290: this information ….“based on current climatic data (1970-2000)”….should better appear under M&M

Response: We repharsed the line “ Our model for current time predicted” and deleted “, which was based on current climatic data (1970–2000)” as this is a general understanding that current layers on Worldclim are from (1970-2000) so we even did not that the time duration in the M&M. Please check in line 301.

Line 291-292: “These predictions are supported by the existing literature” should be deleted (I suggest this option) or completed with citations

Response: We have deleted “These predictions are supported by the existing literature”. Line 303.

Lines 300-302: a bit redundant. I would delete the sentence and start with something like “Both ibex and blue sheep habitats are usually ….”

Response: We have repharsed the lines as “Both Blue sheep and Himalayan ibex habitats are usually between the timber and snow lines at elevations of 3,500–5,500 m, and differ as Blue sheep prefers habitats with steep rolling hills and Himalayan ibex prefer precipitous habitats” Please check 311 to 314.

We have added following phrase to clarify this point (please, see lines 147 - 148).

Further details and information on the realization and interpretation of the WorldClim variables used in this study can be found at <https://pubs.usgs.gov/ds/691/>.

Response: Thank you very much for your precious comments. We have added in the Materials and Methods (please, see lines 147 - 148) the link to the website where the readers can find the modeling and ecological interpretation of all the variables used in this paper. For further detail read “O’Donnell, M.S., and Ignizio, D.A., 2012, Bioclimatic predictors for supporting ecological applications in the conterminous United States: U.S. Geological Survey Data Series 691, 10 p.“ or visit the following website “<https://pubs.usgs.gov/ds/691/>”.

Line 321: add a citation number (not a year) after Aryal et al.

Response: Reference is added as suggested in line 333.

Line 326-327: “or towards a northeast direction” should be either deleted or supported by (at least) a citation.

Response: We have written the indented work and repharsed the lines as “Climate drives evolutionary processes, forcing animals to migrate to higher elevations or extend their distributional ranges towards the Northern Hemisphere [77] or eastward direction [28]” in lines 338 to 339.

Line 329: “began” instead of “begin”

Response: We have made change as suggested in 340.

Lines 346: it would be desirable to have the boundaries of the mentioned NPs outlined on (at least) one of the figures.

Response: We have added boundaries of mentioned national parks to Fig 5 and Fig 6 and also have referred in the text in 362 and 363.

Line 358: “is likely due” sounds better

Response: We have added “likely” as suggested in line 370.

Lines 368-369: I suggest to delete “To do so …… predicted habitats”, since decisions on park boundaries are usually not driven by conservation needs related to one/two mammalian species enjoying a relatively favorable conservation status

Response: We have deleted “To do so” as suggested in lines 379 to 383.
